# “*Don’t wait for them to come to you, you go to them*”. A qualitative study of recruitment approaches in community based walking programmes in the UK

**DOI:** 10.1186/1471-2458-12-635

**Published:** 2012-08-10

**Authors:** Anne Matthews, Graham Brennan, Paul Kelly, Chloe McAdam, Nanette Mutrie, Charles Foster

**Affiliations:** 1 British Heart Foundation Health Promotion Research Group, Department of Public Health, University of Oxford, Oxford, UK; 2 Psychological Sciences and Health, University of Strathclyde, Glasgow, UK; 3 SPARColl, Psychological Sciences and Health, University of Strathclyde, Glasgow, UK

## Abstract

**Background:**

This study aimed to examine the experiences of walking promotion professionals on the range and effectiveness of recruitment strategies used within community based walking programmes within the United Kingdom.

**Methods:**

Two researchers recruited and conducted semi-structured interviews with managers and project co-ordinators of community based walking programmes, across the UK, using a purposive sampling frame. Twenty eight interviews were conducted, with community projects targeting participants by age, physical activity status, socio-demographic characteristics (i.e. ethnic group) or by health status. Three case studies were also conducted with programmes aiming to recruit priority groups and also demonstrating innovative recruitment methods. Data analysis adopted an approach using analytic induction.

**Results:**

Two types of programmes were identified: those with explicit health aims and those without. Programme aims which required targeting of specific groups adopted more specific recruitment methods. The selection of recruitment method was dependent on the respondent’s awareness of ‘what works’ and the resource capacity at their disposal. Word of mouth was perceived to be the most effective means of recruitment but using this approach took time and effort to build relationships with target groups, usually through a third party. Perceived effectiveness of recruitment was assessed by number of participants rather than numbers of the right participants. Some programmes, particularly those targeting younger adult participants, recruited using new social communication media. Where adopted, social marketing recruitment strategies tended to promote the ‘social’ rather than the ‘health’ benefits of walking.

**Conclusions:**

Effective walking programme recruitment seems to require trained, strategic, labour intensive, word-of-mouth communication, often in partnerships, in order to understand needs and develop trust and motivation within disengaged sedentary communities. Walking promotion professionals require better training and resources to deliver appropriate recruitment strategies to reach priority groups.

## Background

Walking has been described as the nearest activity to perfect exercise [1]. Walking at a pace of 5 km/hour expends sufficient energy to be classified as moderate-intensity, defined as 3-6 Metabolic Equivalent Tasks (METs) [2], and contributes to achieving current physical activity guidelines [3]. Indeed the promotion of walking is featured within many international physical activity strategies and national plans [4]. Walking can reduce the risk of all-cause mortality and in particular, cardiovascular disease (CVD) mortality. It also improves diastolic blood pressure (normal range between 60-80 mm Hg) and lipid profiles (a range of cholesterol and triglycerides tests, usually undertaken to assess coronary heart disease risk), both risk factors for CVD and metabolic disease risk factors [5-7]. Regular walking is associated with a reduced risk of type 2 diabetes, reduction in body mass index and body weight, and can improve mood and relieve symptoms of depression and anxiety [8-10]. Increasing overall levels of physical activity by promoting walking will deliver real public health gains via reductions in risk of all-cause mortality [11].

Systematic reviews of the effectiveness of walking interventions found evidence from a range of strategies including brief advice to individuals, remote support to individuals, group-based approaches, active travel (including school based), environmental and community level approaches [12-14]. Indeed, this final strategy was adopted by the large cardiovascular risk reduction programmes of the 1980s which saw the first inclusion of walking promotion in the United Kingdom. In the late 1990s community walking programmes (known as ‘Health Walks’) with designated walk leaders and volunteers, were developed to encourage sedentary adults to become more active. Evaluations of these early projects showed a disparity in the recruitment of different groups. Older active adults were easier to recruit and retain than older inactive adults, with poor health assuming increasing importance as a barrier with increasing age [15]. Other ‘hard to reach’ groups such as families and children, may need greater flexibility in terms of walking programme implementation, given the wide range of participant ages and activity levels [16]. Population levels of walking (as with levels of overall physical activity) remain below recommendations [17-19] and walking behaviour is socially patterned by gender, age, socio-economic status (SES) and type of walking (leisure or transport) [18,20]. These facts readily indicate that the difficulties in walking programme recruitment include not only *how many* but also *who* is recruited.

One criticism of the evidence base for walking interventions is a failure to recruit representative samples of the population. Further studies are needed to broaden the reach of these interventions [12-14] but guidance on achieving this is only partially reflected in public health and clinical research, with the most notable absence relating to conceptual frameworks, procedural models and systems. Indeed research indicates the need to identify what factors are effective in engaging participation at the recruitment phase [21-23]. Further, what is known about recruitment practice relates to drug or medical rather than public health interventions [24], with even less being known about those focusing on physical activity.

The impacts of a walking programme are limited by the efficacy of dose (how well does the intervention works on its participants) and also by recruitment (maximising the numbers of participants from the target populations who will receive the intervention dose). The Scottish Physical Activity Research Collaboration (SPARColl) (http://www.sparcoll.org.uk) has piloted a series of studies to examine the effectiveness of different recruitment strategies for community based programmes of walking promotion. We defined recruitment for such walking studies or programmes as *‘the process of inviting participation to a formal activity including the invitation, informing and facilitation of interested parties to take part in an organised study, activity or event.’* This paper examines the experiences of walking promotion professionals and the range of recruitment strategies adopted by community based walking programmes within the United Kingdom, and discusses their views of effectiveness of such strategies in relation to particular population groups.

## Method

### Research team

The research was undertaken by two members of the research team, one a female doctoral qualitative researcher of 15 years experience and the other a male Ph.D candidate. No relationship was established with the participants prior to research commencement, but on initial contact participants were provided with some background to the researchers and the research groups to which they belonged. The research team had a long-standing interest in physical activity recruitment, latterly focusing on walking as an area needing research development. The proposed research was awarded ethics approval from the University of Strathclyde Ethics Committee in March 2009.

### Design

The aim of the research was to identify the experiences of walking promotion professionals on the range and evidence of effectiveness of different recruitment strategies to encourage adults and children to participate in walking promotion projects. In addressing this aim the research adopted a phenomenonological theoretical orientation, practiced through qualitative techniques. Such an approach seeks to study actions, situations and the realities constructed within particular spheres of human life [25].

### Sampling

In seeking to gather data from those likely to possess the greatest knowledge and experience of walking programme recruitment the study employed purposive sampling, where the sample units possess particular characteristics which enable detailed exploration of particular issues [26]. Three walking promotion agencies (to be known as Agency A, Agency B and Agency C) were chosen to provide research participants – all agencies had national representation across England and Scotland, were experienced in providing community walking programmes, and possessed personnel (either paid or volunteer) with specific responsibility for programme recruitment. Senior managers from each agency were contacted by telephone. Information about the research was shared and all three agencies agreed to their involvement. The senior managers forwarded a list of personnel based on the following criteria pertinent to the research: likely to agree to participate, working at local level, managing or organising walking programmes, and having direct responsibility for recruitment.

Potential participants on all contact lists were either emailed or telephoned (if no email address) with a research outline and an invitation to participate. Of thirty-seven contacts, eight failed to respond and one felt they could not participate as they were not involved with recruitment currently. Twenty eight participants were drawn into the sample, spread across the UK: 5 from Scotland, 7 from the North West, 1 from Yorks and the Humber, 4 from the West Midlands, 2 from the East Midlands, 1 from London, 5 from the South West and 3 from the South East. Table 1 shows how the 28 walking programmes were distributed by aim and target population.

**Table 1:** Walking programmes by aim and target population

**Target population**	Agency A*	Agency B / Agency C*	**Other**	Programmes with health aims**	**Programmes with no health aims**
				**Total (n = 21)**	**Total (n = 7)**
Open to all	6				**6**
Aged 20-40	1				**1 (case study A)**
Children and families	2			**2 (inc. case study B)**	
Aged 55+		1		**1**	
Sedentary	2	15		**17 (inc. case study C)**	
Mental health groups			1	**1**	

* Agency A (England) primarily promotes walking.

* Both Agency B (England) and Agency C (Scotland) primarily promote walking for health.

** Programmes which specifically seek to improve the health of individuals.

### Data collection

Data collection adopted two techniques: telephone interviews (conducted with participants in their workplaces or homes), and case study participant observation [27] with face to face interviews (conducted in the field). Telephone interviews, an approach commonly adopted when budgetary restrictions limit travel and time [28] were undertaken using a data collection tool containing standard questions developed to facilitate systematic data gathering. Included in this tool were questions addressing the purpose and structure of walking projects, participant target groups, recruitment method selection, successes and failures, participant retention, and recruitment evaluation. The interviews were audio-recorded and were semi-structured, an approach useful for gathering facts, identifying motives, commenting on actions and eliciting reasons and explanations [29]. Field notes were made during and immediately after interviews. By the end of the telephone interview round it was felt that data saturation had been achieved.

Since the aim of the research was to explore the issue of recruitment in the greatest depth, case studies (which provide data facilitating deeper understanding of aspects of routine and culture), were included on completion of an initial review of participant programmes. Three were selected on the criteria of working with hard-to-reach groups and also demonstrating innovative recruitment methods. Informal, semi-structured and group interviewing alongside participant observation of project leaders and walkers provided a richer understanding of the ‘experience’ of recruiting [30], from the viewpoint of both the recruiter and the recruitee.

### Analysis

**Figure 1:**
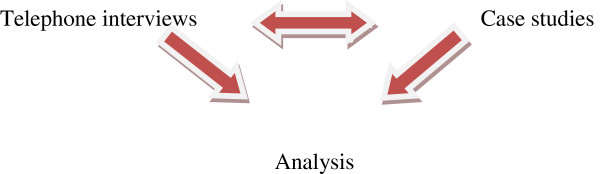
Flowchart showing analysis of data.

Exploratory research, such as this study which seeks to find out what recruitment methods are adopted by walking recruiters, may draw on multiple strategies. For example, case studies *within* surveys serve to enable the researcher to answer ‘how’ or ‘why’ questions [31]. As such, strategies are not seen as mutually exclusive but as providing complementary explanation to the issues explored, and therefore contributing to the theory building process (see Figure 1).

**Figure 2:**
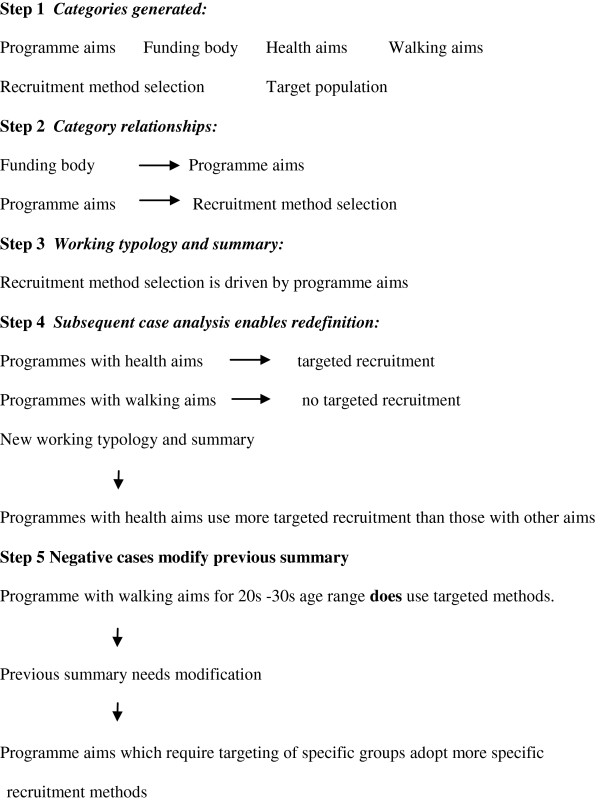
Example of analysis using analytic induction.

Data analysis adopted an approach using analytic induction. Such an approach, common to qualitative research, proceeds through the following series of steps [32]: (i) data are scanned to generate categories of phenomena, (ii) relationships between categories are sought, (iii) working typologies and summaries are written on the basis of the data examined, (iv) subsequent case analysis enables refinement and redefinition, and (v) negative and discrepant cases are deliberately sought to modify, enlarge or restrict the original explanation or theory. Using the Excel software package, the data were coded in the first instance by one researcher, followed by a cyclical pattern of thematic verification and revision during analysis with two other researchers (see Figure 2 for an example of this ‘step’ procedure in practice).

## Results

### Programme aims: why purpose drives recruitment

Key drivers for recruitment are the aims and objectives of programmes. In general, the data indicate that the less programmes are driven to capture specific populations, the less targeted the recruitment drive. Health walks programmes – ‘specifically designed and carried out to improve one's health’ [33] – seek out particular population groups based on health criteria. ‘Walking-focused’ programmes (on the other hand, as typified by those run by Agency A, seek out individuals based on walking criteria. Although as a subscription-driven walking charity Agency A now cites the promotion of social welfare (incorporating health) within its aims, historically it was formed to promote access to the countryside and the ‘right to roam’. These are aims which still predominantly define the way open walking programmes are structured within the organisation.

"the problem they have is, some of their traditional members have a lot of discomfort with promoting the urban walking agenda…I think they see it as detracting from the core stuff about footpaths…"

"Respondent 5, Case study A (Agency A)"

Data from the six Agency A walking leaders who provide ‘open to all’ programmes, showed that the typical demographic of recruited members is white, middle class, and retired. Given that such programmes are not seeking to recruit from specific groups, non-targeted recruitment methods are typically employed, e.g. placing promotional material in community spaces and/or local media in order to engage anyone interested in walking from the population at large.

Data from the health walks programme leaders on the other hand, show recruitment strategies driven in part by health aims. Typically programmes had pre-identified target groups (e.g. sedentary people, people living in areas of deprivation) defined within their aims, which led to targeted recruitment strategies. Although most respondents did mention the use of promotional material, it was recognised that this type of recruitment would not engage those for whom lifestyle changes were a necessary precursor.

"To me that’s not the target group that we want [those that read posters] because obviously if they’re out and about they’ve seen them anyway, so they are getting out and about. We prefer to try to get to the people that need to walk."

"Respondent 17, (Agency B)"

Commonly adopted strategies were clearly linked to working in face to face contexts.

"Our mission statement is to get more people more active more of the time…When you’re targeting a certain group of people [i.e. the inactive] they’re not going to read something in a health and fitness magazine, they’re not necessarily going into a leisure centre to pick up information from there. So rather than waiting for them to come to us, we’re going to them."

"Respondent 15, (Agency B)"

The majority of respondents who worked within programme aims which targeted ‘hard to recruit’ groups e.g. Black and Minority Ethnic (BME) or vulnerable children and family groups, mentioned the need to work in partnership with those organisations and agencies currently working with those groups.

"[Agency A have] not really [got] the expertise or contacts to do it with children or families. So we needed help in that direction and [named children’s agency] have got that national spread of working with children and contact through family centres throughout the country”"

"Respondent 3, Case Study B (Agency A)"

In terms of actually recruiting to walking programmes those from ‘hard to recruit’ groups who had never walked, there was a recognition that engagement is largely achieved through the trust and motivation which partner organisations build up with their clients. Recruitment at this level was typically observed as inter-personal – intensive face-to-face, word-of-mouth prompting.

"Walk leader: I don’t know if it would have been possible [to run the programme without the help of a partner], because all the BME groups that I’ve run, I’ve always had some sort of community worker attached to them… [without that] they probably didn’t know who Agency A were, they wouldn’t have heard of [named walking programme]."

"Community Programme Manager: …my Assistant has worked her socks off to get them women…The day before you’re ringing, you’re sending letters out, ringing the morning before, you’ve got to really motivate [the Asian women to participate in the walking sessions]…"

"Group interview, Case Study C, (Agency A)"

To summarise this finding, programme aims which require targeting of specific groups adopt more specific recruitment methods.

### Recruitment processes: what guides the activity of recruitment?

A key principle which guides recruitment is the conceptual framework, if any, underpinning each programme. Of the 28 walking programmes, only 5 (all with health aims) were working within a conceptual framework which established recruitment strategy. Of these, 3 programmes were staffed by paid coordinators, where the remit was to work with partner organisations and community groups to reach ‘hard to recruit’ groups. These were guided by the adoption of actively targeted approaches as described above.

Two other programmes operated under theoretical models which informed the recruitment process. One was a health walks programme guided by the Stages of Change model [34], which led to the provision of walking interventions of differing intensities designed to cater for the needs of the local population at differing stages of engagement. Here, longevity was perceived as facilitating effective ‘word of mouth’ recruitment. The other, a walking programme run by a drug addiction team, operating under a bio-psychosocial model [35] to bring about mental and physical health improvement, used ‘word of mouth’ recruiting firstly via therapy sessions and encouraging clients to bring others. These programmes indicate at least some kind of formative evaluation through participatory planning with other stakeholders [36]. Data from the remaining 23 programmes indicated the decision about which recruitment methods to adopt was largely the responsibility of walking programme coordinators, who operated under no guiding conceptual framework.

**Figure 3:**
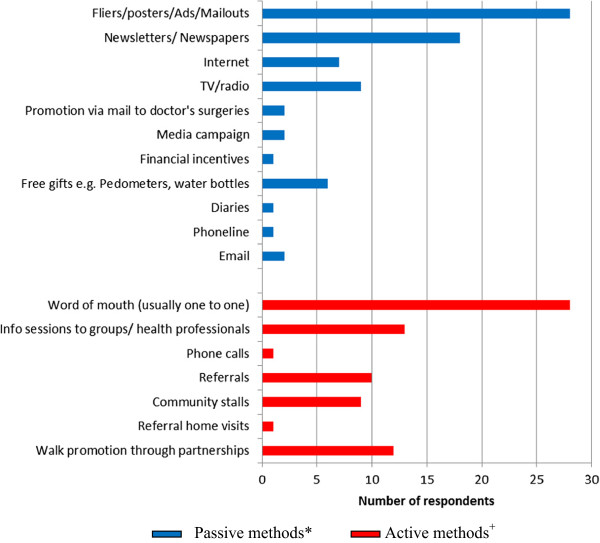
Recruitment methods used by projects.

Having established that most respondents had no conceptual framework to guide their recruitment activity, we now examine which recruitment methods were selected. Figure 3 categorises the methods according to two types of recruitment approach – ‘passive’ and ‘active’.

**Figure 4:**
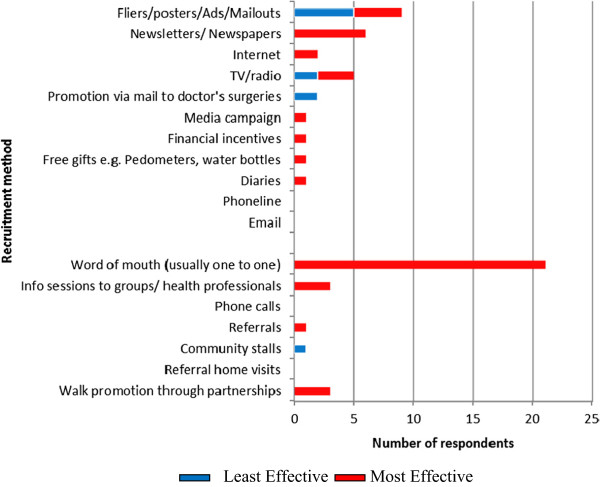
Effectiveness of recruitment methods used.

Passive methods are defined as ones which require the potential programme participant to make the first contact with the programme. Active methods are defined as ones which require a programme representative to make the first contact with the potential participant. The data show a fairly even distribution between passive and active methods used, although the only active method used by ‘open to all’ programmes was ‘word of mouth’. Programme leaders were then asked to nominate the method thought to be the most and least effective. Figure 4 shows that active methods – particularly ‘word of mouth’ – were overwhelmingly believed to be the most effective.

Despite the popularity of the use of fliers and posters shown in Figure 3, only a small number of programme leaders believe them to recruit effectively, with some regarding them negatively. The data seem to indicate a mismatch between the methods respondents believe to be *effective* and the ones they actually *adopt*. Other than the ‘open to all’ Agency A groups, who typically used the fewest number of recruitment methods (typically programme fliers and word of mouth), the majority of respondents, none of whom were guided by presence of a conceptual framework, used a ‘belt and braces’ approach encompassing as many methods as their capacity allows.

"Everything can work. You just have to try everything."

"Respondent 27, (Agency C)"

Three respondents with a personal background in marketing were identified during interviewing. Notable was their adoption of ‘what works’ recruitment, all favouring active ‘word of mouth’ community-based approaches. In this sense their recruitment activity appeared to be more strategic than many other respondents, where the start-point focused on recruitment *outcomes* (how best to recruit the target group) not recruitment *processes* (how many potential recruitment methods could be employed). In explaining why respondents were so process- rather than outcome-focused in their recruitment, the data indicated the influence of resource availability. Although respondents thought of active recruitment methods as more effective, they described them as time-intensive and draining of human resources.

"…we have no budget for advertising or promotions or anything. It was purely salary-based…We’re doing a lot of social and community promotions…Which is actually quite labour intensive"

"Respondent 15, (Agency B)"

Of the 28 programmes, none worked within a specific recruitment budget, although a few possessed a ‘publicity’ budget. Funded post respondents commonly spoke of recruitment being under-resourced. For example, those programmes which fell under the Agency B umbrella – typically gathering meagre funding from multiple sources – tailored to their recruitment methods according to resource capacity which drove them to adopt the cheapest methods.

"Yes, it is NHS [National Health Service] funding, if you classify my wages as funding. Other than my time to coordinate the scheme, the other funding pot comes from the [named] Council. They will print my timetable for me but then it’s down to me to distribute that. There’s no other funding set aside for the walking scheme as such…with regards to targeting the population groups and the ways in which we do it, because I’ve got no budget I’m restricted to leaflets…"

"Respondent 18, (Agency B)"

To summarise this finding, without the guiding principles of a conceptual framework for recruitment method selection, very few recruiters are strategic in thinking of ‘what works’. Most focus on multiple method selection processes often driven by resource-poor contexts.

### Sustainability: the contribution of evaluation and training to recruitment

#### Evaluation – planning and measures of success

A key component of sustainability is effective programme evaluation. There was a wide variation in the degree of evaluation found. Twenty-seven of the 28 interviewees engaged in some kind of ‘process’ (assessing implementation) evaluation. Of these, 5 had also either been or were about to be evaluated by independent researchers. It was clear that the vast majority of evaluations focused on evaluating participation with none methodically collecting exposure, delivery or context data. All programme evaluations noted numbers of participants, a measure of primary importance.

"We do count up how many people we get on a walk. I wouldn’t say there was competition exactly [between walk leaders], but if you get 30+ the leader gets a certain smugness!"

"Respondent 10, (Agency B)"

In the case of health walk programmes run under the Agency B umbrella, evaluations were usually based on data obtained from the Outdoor Health Questionnaire (OHQ) which walkers complete on their first visit. Questions about method of recruitment, health status, background as a walker, and weekly engagement in moderate levels of physical activity, are collated at local level by coordinators and at national level by Agency B. No respondent reported interrogating the OHQ data to relate numbers of participants to their health or physical activity status, and therefore whether or not they were representatively capturing the ‘sedentary’ target populations they sought to recruit. Thus, despite respondents’ awareness of the target population, the focus of most evaluation seemed to be *descriptive* rather than *diagnostic* in terms of prospective recruitment direction. As shown by Figure 4, respondents believed the most effective recruitment method to be ‘word of mouth’, often quoting their OHQ data as evidence, but no programme evaluated recruitment methods used and their relative success in achieving a representative sample population.

"We do have a database and record how many participants there have been to the programme, but we don’t necessarily relate that to whether we’ve just delivered a load of leaflets or whatever."

"Respondent 13, (Agency B)"

Other than one respondent (whose programme measured Body Mass Index ‘before and after’ no programme evaluated outcomes (treatment effectiveness) systematically, although anecdotal evidence was widely offered. Across all programmes, it was the *number of participants* and the *level* of recruitment, i.e. process evaluation data, which was used and accepted as evidence of effectiveness instead.

"We feed into Agency B’s database now…what I’m able to use if for now is the number of new walkers. I can certainly track every single walker now to see how regularly they are walking. We have a Service Level Agreement…I have to report the number of walker attendances. The expectation is that that will have increased."

"Respondent 16, (Agency B)"

#### Training – knowing how to recruit

The Agency A Membership Recruitment and Publicity Handbook [37] lists ideas for recruitment, but local groups are advised to consider using a whole range of methods in a ‘belt and braces’ approach. Word of mouth is encouraged as a successful method, but is phrased as ‘recommendation to a friend’, a suggestion likely to succeed only insofar as recruiting those from similar backgrounds. Agency B and Agency C now embrace health-related social marketing, defined as

*"‘the systematic application of marketing, alongside other concepts and techniques, to achieve specific behavioural goals, to improve health and reduce health inequalities’. *[38]"

In thinking carefully about the barriers to walking for particular groups [39], the skill of the publicity officer in Case Study A, who came from a marketing background, was in matching the nature of the group to the motivations of the 20s to 30s audience.

"We unashamedly did a Valentine’s feature this year, because it is like a dating club, our group."

"Respondent 5, Case study A (Agency A)"

In a contrast to all other programmes, fliers and posters were not adopted by Case Study A as recruitment tools, although word-of-mouth between friends was still cited as important. Innovative recruitment practices included the use of new social communication media – the group used the internet exclusively to communicate (and recruit) via their website, Time Out, Facebook and Twitter – including ‘piggybacking’ walking onto existing events e.g. ‘Films on Foot’, walking to well-known film locations as part of a city film festival. Such strategies thus tapped into the cultural norms and behaviours of young urban populations, presenting walking as ‘cool’ and therefore appealing.

Under social marketing principles, recruiters of health walks should emphasise social rather than health benefits, a strategy thought to be more persuasive. Indeed one interviewee alluded to these potential ‘negative’ perceptions of promoting ‘health’.

"I don’t advertise it as a health walk any more. All my promotion says, ‘get out, make friends, have fun,’…just telling them the walk will do them some physical good won’t necessarily motivate them…the social aspect is much more motivating."

"Respondent 13, (Agency B)"

Indeed this social aspect of walking programme sustainability was emphasised by many respondents, particularly in relation to combating social isolation [40], believing that participants are retained on programmes by the interactions they have with volunteers and fellow walkers.

"…participants pick up that enthusiasm from the volunteers…Most of the time people are going on the walks because they want a chat…[volunteers] listening to the stories if someone’s been poorly or something’s happened to someone in their family."

"Respondent 20, (Agency B)"

Such ‘easy’ retention, perhaps amongst programmes attended by long-standing walkers, may not be the experience of those trying to recruit in more challenging settings. Three research case studies, working with ‘hard to recruit’ groups, richly demonstrated that recruiters working with groups that *don’t already* walk need to understand what will *persuade* them to walk. However, the interview data show that most recruitment decisions are taken by programme coordinators ‘on the ground’, often piecemeal, none of whom have received any formal recruitment or marketing training in effectively reaching the target population. Therefore, whilst it is clear that some walking organisations have embraced the need to market persuasively to the inactive [41] and now offer social marketing training courses [40], the data here show gaps in the effective delivery of that training which might help to facilitate sustainable walking programmes for targeted groups.

To summarise this finding, sustainability seems dependent on *how many* rather than *which* participants are recruited to walking programmes. Recruiters know from experience how to retain participants who are already committed to walking programmes, but do not receive standard training to help them recruit the correct participant representation at the outset.

## Discussion

We successfully identified a range and views of the effectiveness of different recruitment strategies to encourage adults and children to participate in walking promotion projects, from the experiences of walking promotion professionals. Our research has findings which indicate three key messages.

1. Walking programmes with aims which necessitate recruiting specific groups seem to adopt ‘targeted’ recruitment methods; participants perceive such methods as the most effective in engaging ‘hard to recruit’ groups.

2. Weak programme structures, including the lack of conceptual frameworks and resources, may lead recruiters to focus on less expensive but potentially less effective recruitment processes.

3. Sustainability seems dependent on *how many* rather than *which* participants are recruited to walking programmes. Recruiters know from experience how to retain participants who are already committed to walking programmes, but do not receive standard training to help them recruit the correct participant representation at the outset.

The finding that the aim of a walking programme directly influences the recruitment framework, demonstrates the inadequacy of a ‘one size fits all’ approach to walking programme recruitment. For programmes that are walking-focused, such as the traditional volunteer-led programmes offered by Agency A, 72% of whose membership consists of professionals, there is no surprise that they tend to recruit from similar demographic groups – the retired, middle class, largely female constituency.

However, it has been suggested that walking as an activity in England is decreasing [42], a trend reflected in the falling membership of Agency A. A recent Agency A strategy document advocates the need to ‘*Build a more diverse supporter base, less dependent on those retired or approaching retirement*’ [43]. This aspiration is clearly a challenge at local level where the recruitment work is undertaken through the goodwill of untrained volunteers, not equipped to adopt the community-based targeted messaging approaches which Agency A recognises is a necessary strategy to engage inactive groups in walking [44].

It has already been shown that health walks tend to recruit people who already walk, who are already reasonably fit [45], and that ‘new’ walkers from disadvantaged groups form a small percentage of health walks in general [15]. The finding here, that health walks programmes can recruit successfully by adopting more ‘active’ approaches, demonstrates an acceptance that walking interventions may present barriers to specific groups which require an understanding of individual needs, as reflected in recent trends endorsing social marketing techniques. Walking interventions have the potential to increase physical activity levels at least in the short term. This research indicates the importance of recognising the connection between recruitment to any given walking intervention and the life circumstances of potential participants.

Beyond the finding that coordinators recruit in specific ways because of programme aims, the data seems to indicate that recruitment method selection is affected by two other factors: awareness of effectiveness and financial capacity. For those programmes where there was a paid coordinator in post, of interest is the finding that despite perceiving ‘word of mouth’ as the most effective method, the adoption of a range of methods, including those perceived to be the least effective, predominated. Coordinators with a background in marketing were notable for not taking this approach; instead selecting methods strategically based on their perceived effectiveness. It is therefore suggested that a necessary precursor to effective recruitment is personnel training, a finding which carries financial implications for programme funders. ‘Word of mouth’, at its most effective when face-to-face [46], is labour intensive when compared with other recruitment methods. Programme coordinators privilege some recruitment methods over others because of budgetary constraints. This research indicates that weak programme structures lead to weak recruitment processes, a message which may be opaque for funders since, irrespective of the original target groups, recruiters are indeed successful at drawing participants into walking programmes.

Previous research has indicated the paucity of research examining the effectiveness of community-based physical activity interventions which operate by tapping into existing interpersonal links [47]. This research indicates that such interventions will be more likely to succeed in recruiting and retaining the target population effectively, only by building the capacity to deliver adequate and standard training.

Good process evaluation should examine key elements – programme exposure, participation, delivery and context [36]. For the most part, programmes undertook fairly basic process evaluation, largely concerned with participation rates. The finding that programmes do not evaluate recruitment method effectiveness may in part reflect the hand-to-mouth nature of some programmes. For many coordinators, the on-going struggle for financial sustainability and survival could explain the focus on numerical attendance, rather than on who is being recruited and how. More widely, this may mean that programmes without evaluation to reveal the ongoing representativeness of the target population within the sample, will be unable to demonstrate their true effectiveness, despite their popularity in general.

This study of walking recruitment has certain limitations. There were the usual research constraints of time and money. It therefore confined its respondents to those from only three organisations which promote walking, and limited the number of respondents. In addition, the respondents’ experiences of recruitment are unverified by any other sources and may be weakened by ‘telephone’ rather than ‘face-to-face’ interviewing. The small and un-representative nature of the sample also limits the extent of generalisability. However, this area of enquiry is under-researched and therefore these findings provide a useful background to understanding what constitutes effective walking recruitment methodology in relation to sedentary populations.

## Conclusions

Ogilvy et al (2007) warn that targeted walking interventions may preferentially be taken up by better-off groups and may therefore have the potential to increase health inequalities [12]. Indications from this research resonate with similar anxieties – effective walking programme recruitment, as perceived by recruiters, seems to require trained, strategic, labour intensive, word-of-mouth communication, often in partnerships, in order to understand needs and develop trust and motivation within disengaged sedentary communities. Participants from such communities seem often to be the hardest to recruit but nevertheless are often those who stand to benefit the most. Unless this issue is recognised by policymakers, inadequate resources may compromise the sustainability of effective walking programme recruitment processes for such target audiences.

## Competing interests

The authors declare that they have no competing interests.

## Authors’ contributions

CF, GB & AM conceived of the study, and participated in its design and coordination and helped to draft the manuscript. PK collected data and helped to draft the manuscript. CMcA and NM helped study coordination and drafting the manuscript. All authors read and approved the final manuscript.

## Pre-publication history

The pre-publication history for this paper can be accessed here:

http://www.biomedcentral.com/1471-2458/12/635/prepub
